# Large-scale extraction of gene interactions from full-text literature using DeepDive

**DOI:** 10.1093/bioinformatics/btv476

**Published:** 2015-09-03

**Authors:** Emily K. Mallory, Ce Zhang, Christopher Ré, Russ B. Altman

**Affiliations:** ^1^Biomedical Informatics Training Program, Stanford University, Stanford, CA 94305, USA,; ^2^Department of Computer Sciences, University of Wisconsin-Madison, Madison, WI 53706, USA,; ^3^Department of Computer Science,; ^4^Department of Bioengineering,; ^5^Department of Genetics and; ^6^Department of Medicine, Stanford University, Stanford, CA 94305, USA

## Abstract

**Motivation:** A complete repository of gene–gene interactions is key for understanding cellular processes, human disease and drug response. These gene–gene interactions include both protein–protein interactions and transcription factor interactions. The majority of known interactions are found in the biomedical literature. Interaction databases, such as BioGRID and ChEA, annotate these gene–gene interactions; however, curation becomes difficult as the literature grows exponentially. DeepDive is a trained system for extracting information from a variety of sources, including text. In this work, we used DeepDive to extract both protein–protein and transcription factor interactions from over 100 000 full-text PLOS articles.

**Methods:** We built an extractor for gene–gene interactions that identified candidate gene–gene relations within an input sentence. For each candidate relation, DeepDive computed a probability that the relation was a correct interaction. We evaluated this system against the Database of Interacting Proteins and against randomly curated extractions.

**Results:** Our system achieved 76% precision and 49% recall in extracting direct and indirect interactions involving gene symbols co-occurring in a sentence. For randomly curated extractions, the system achieved between 62% and 83% precision based on direct or indirect interactions, as well as sentence-level and document-level precision. Overall, our system extracted 3356 unique gene pairs using 724 features from over 100 000 full-text articles.

**Availability and implementation:** Application source code is publicly available at https://github.com/edoughty/deepdive_genegene_app

**Contact:** russ.altman@stanford.edu

**Supplementary information:** Supplementary data are available at *Bioinformatics* online.

## 1 Introduction

A complete repository of the gene–gene interactions is a key for understanding cellular processes, human disease and drug response. Furthermore, these interactions inform gene network analyses that typically rely on curated interaction databases. Two types of interactions are critical for understanding how a protein or gene affects biological or disease processes: physical protein–protein interactions (PPIs) and transcription factor interactions (TFIs). PPIs include interactions where two proteins physically bind to one another to form a complex or otherwise modify the function of one or both proteins. Alternatively, TFIs involve transcription factors directly binding upstream of a gene to control transcription of that gene. Modifications to PPIs and/or TFIs can have a detrimental effect on their associated cellular processes. An improved understanding of both of these interactions (henceforth called gene–gene interactions for simplicity) may help uncover the genetic basis of many complex diseases and the impact of small molecules (including drugs) on these networks.

Repositories such as BioGRID (Chatr-Aryamontri *et al.*, 2014) (http://thebiogrid.org/), the Human Protein Reference Database (Keshava Prasad *et al.*, 2009) (HPRD; http://www.hprd.org/) and the Database of Interacting Proteins (Salwinski *et al.*, 2004) (DIP; http://dip.doe-mbi.ucla.edu) contain high-quality curated physical PPIs. Additionally, the ChIP Enrichment Analysis database (Lachmann *et al.*, 2010) (ChEA; http://amp.pharm.mssm.edu/chea/chipchip.php) provides both curated and high-throughput transcription factor relationships. However, these and other resources require manual curation of the biomedical literature to gather gene–gene interactions. While biomedical curation is valuable, literature repositories, such as the National Center for Biotechnology Information’s PubMed database, continue to grow exponentially (Larsen and von Ins, 2010). As of March 2015, PubMed contained over 24 million citations and over 15 million abstracts. Complete and constant curation of such a large and continuously growing database is costly, both in terms of time and money. As the literature continues growing, resources such as BioGRID, HPRD and DIP may miss gene–gene interactions. Similarly, human error may introduce false relationships depending on the definition of an interaction and the associated source. Thus, although these resources contain easily accessible high-quality relationships, they cannot be treated as the sole sources of all known gene–gene interactions.

The growing burden on manual curation has spurred the development of text mining tools for biological entities and relations (including protein interactions). The simplest approach for text mining relations is entity co-occurrence where a relationship between two entities is proposed based on sentence- or abstract-level co-occurrence statistics. While useful for well-established relationships, these approaches tend to introduce a large number of false positives if no filtering technique is applied. Furthermore, they cannot detect low frequency relationships. The STRING database (Franceschini *et al.*, 2013) uses protein–protein co-occurrence as one type of text mining evidence for protein interactions; however, this type of evidence tends to have a lower weight compared with other types of PPI evidence in STRING. PPI Finder extracts protein interactions using co-occurrence at the abstract level along with relevant interaction words and PPI database and Gene Ontology term matches (He *et al.*, 2009). Other methods identify articles or sentences that contain PPIs but do not focus on the precise interactions (Chen *et al.*, 2014; Hoffmann and Valencia, 2004; Kim *et al.*, 2012). Still other systems use rules to extract interactions; PPLook (Zhang *et al.*, 2010) and PPInterFinder (Raja *et al.*, 2013) use specific interaction patterns to extract PPIs from sentences. These high precision patterns include ‘A interact with B’ and ‘Binding of A and B’. While rule-based approaches can achieve high precision, their rules can reduce recall. Unsupervised and semi-supervised approaches allow for scalability and look for novel patterns in a new document corpus. To address these different types of systems, Quan *et al.* (2014) developed both an unsupervised and semi-supervised system for biological entity extraction (including PPIs) that uses interaction pattern clustering, dependency parsing and phrase structure to extract interactions from text. Despite these and other contributions (Czarnecki *et al.*, 2012; Papanikolaou *et al.*, 2015; Tikk *et al.*, 2010), there remains a need for a high performing semi-supervised scalable text-mining tool to create a more complete gene–gene interaction database.

Text mining systems are designed to extract information from text in a domain-oriented manner. DeepDive is a trained system for extracting information from a variety of sources, including text (Niu *et al.*, 2012a, b). The name DeepDive refers to the system framework that includes application code and an inference engine. While DeepDive provides the inference engine, users write the application code for their specific task. This application code includes entity or relation definitions, feature generation and training example labeling. Users also write the inference rules used by DeepDive during the inference. These probabilistic rules include features and rules describing relationships between entities or relations.

Specifically for text extraction, the pipeline for DeepDive includes (i) text preprocessing, (ii) candidate entity or relation construction, (iii) inference and (iv) system tuning. Text preprocessing includes parsing the full text for sentences, tokens or words, parts of speech and dependency graph relationships. Extractors in the application take these sentences as input and construct candidate entities or relations that include features and labels for training examples. The inference engine in DeepDive uses these candidate mentions and relations, along with inference rules, to compute probabilistic predictions for each candidate. This system relies on human experts to tune specific entity and relation extractors by developing useful features and training examples for their specific use case. DeepDive has been successfully applied to two scientific domains (Peters *et al.*, 2014; Zhang *et al.*, 2013), but biomedical text and entities provide a unique challenge due to the ambiguity of many entity names and shared context of many co-occurring genes.

DeepDive’s underlying probabilistic graphical model allows users to automatically label training data to learn important features. This automatic labeling is done using distant supervision (Mintz *et al.*, 2009). This labeling is called distant supervision because the user automatically labels a set of training data using known entities or relationships from an independent data source. In this way, the user can apply distant supervision to quickly scale the training data without need for manual labeling. Recently, text mining methods have begun using distant supervision for specific biological domains (Poon *et al.*, 2015), but distant supervision has not been used for large-scale labeling of gene–gene interactions from either biomedical or biological context.

Although current PPI text mining methods have begun leveraging expert opinions to extract information from text (Tastan *et al.*, 2015), these methods have not been developed for a large scale extraction task. With this in mind, we aimed to extract gene and protein interactions from text using DeepDive and expert tuning. Additionally, we applied the concept of distant supervision to biomedical text mining without limiting the application to a specific process or pathway. Finally, we applied this system to the entirety of three PLOS journals to extract all known human gene–gene interactions.

## 2 Methods

**Fig. 1.**
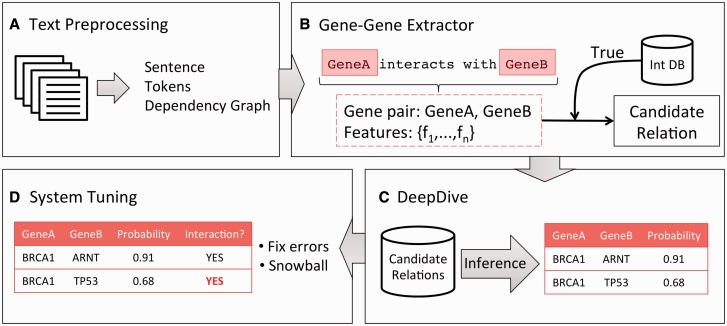
Gene–gene extraction pipeline. (**A)** We performed text pre-processing to parse documents into sentences and tokens and to construct dependency graphs between tokens in the sentences. This parsed data were stored in a sentences database. (**B**) The gene–gene extractor constructed candidate relations from the sentences and deposited them into a database. These relations composed of a pair of genes and features from the sentence. (**C**) DeepDive calculated probabilities that the candidate relation was an interaction using inference rules based on the features. (**D**) We performed system tuning to identify and correct system errors. Furthermore, we performed a snowball technique where we input correct relations as new training examples in the next system iteration

An overview of the gene–gene extraction system is depicted in Figure 1 and is described below. First, we parsed a corpus of documents for sentences, words (or tokens) and dependency graphs. Next, the gene–gene extractor constructed candidate relations from the parsed sentences. These candidate relations consisted of two co-occurring genes in the sentence and the set of features that describe their relationship based on the text. After we marked a set of these relations as training examples, DeepDive calculated a probability that the candidate relation was a true gene–gene interaction in the sentence. Finally, we performed a tuning process to increase system performance.

### 2.1 Literature corpus

We obtained full-text PDF documents from *PLOS One, PLOS Biology* and *PLOS Genetics* (Zhang and Re, 2015). We chose the journals due to their relevance to biology and genetics research from the PLOS publisher. These corpora contained 102 764, 3565 and 4416 documents, respectively. We converted each PDF document to text using the Tesseract OCR engine (Smith, 2007), and we parsed the text of each document using Stanford CoreNLP 1.3.4 (Manning *et al.*, 2014). Each document was split into individual sentences and each sentence tokenized. Tokens were labeled with parts of speech tags, named entity recognition tags and dependency graph relationships to other tokens in the sentence.

### 2.2 Gene–gene extractor

The gene–gene extractor takes a document or sentence as input and constructs a set of candidate relations. Here, a candidate relation has two components: two co-occurring genes in the sentence and the set of features that describe that gene pair in the sentence. We will refer to both proteins and genes as genes unless discussing a specific type of interaction (e.g. a physical PPI).

#### 2.2.1 Extracting co-occurring gene pairs

We define a co-occurring gene pair as two genes co-occurring within a sentence in a given document. We restrict sentences to contain no more than 50 tokens. The extractor annotated tokens in the sentence as genes if they occurred in a dictionary of human gene symbols containing both HUGO Gene Nomenclature Committee (HGNC) official symbols and alternate symbols from the NCBI Gene database. We require genes to match the dictionary exactly and do not accept case-insensitive matches. Finally, we only extract gene symbols as a gene mention and disregard full name mentions. From the set of annotated gene tokens in a sentence, we constructed all pairs of distinct gene symbols.

#### 2.2.2 Extracting features

**Fig. 2.**
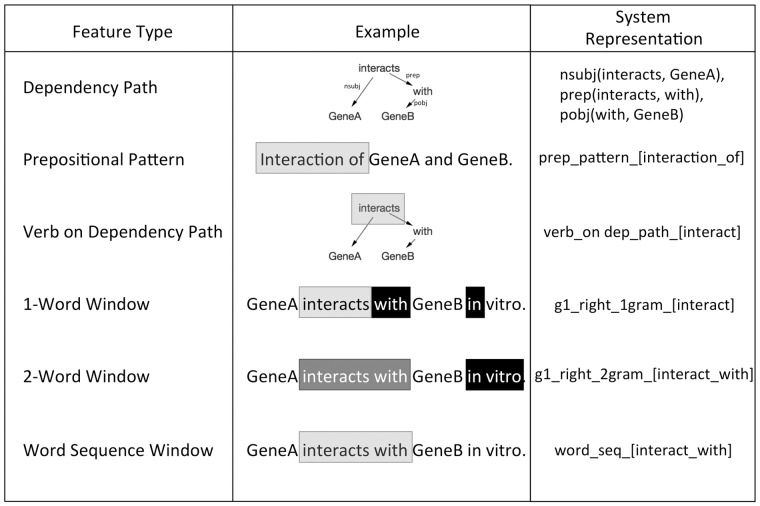
High-level feature patterns. Boxes represent relevant patterns in the sentence for the feature. Light gray boxes indicate features for GeneA and black boxes indicate features for GeneB. Shared boxes are represented with a medium gray box. The feature applies to both genes if only a light gray box is present

Each candidate relation included a set of features that provided textual information about the co-occurring gene pair from the associated sentence. Feature categories and examples are depicted in Figure 2. These features included one and two word windows around each gene entity in the sentence, prepositional interaction patterns and a variety of dependency graph features. Instead of hard-coding specific features before running the extractor, we developed high-level feature patterns that became specific binary features based on the input sentences. For example, instead of specifically creating a feature for the phrase ‘interacts with’ between the two genes in the sentence, we created a high-level feature pattern for any word sequence occurring between the two genes in the sentence. In this way, we developed a binary feature for ‘interacts with’ between genes and binary features for any phrase occurring between the genes in the training data.

### 2.3 Distant supervision

We used distant supervision (Mintz *et al.*, 2009) to apply labels to gene–gene candidate relations. Distant supervision allows us to apply a label (True, False or Unknown) to a candidate relation if the interaction status of the gene pair is known from an independent source. Without distant supervision, DeepDive does not know that the candidate relation is a true interaction. However, if we know that two genes interact from an independent source, we can label all sentences appropriately and use these labeled candidate relations as training examples.

We created an is_correct variable for each gene–gene candidate relations and labeled is_correct as True (known PPI or transcription factor relationship), False (known negative interaction or randomly selected candidate relation) or Unknown. We labeled candidate relations as True if the relations occurred in the BioGRID database with Co-crystal Structure, Reconstituted Complex or Co-purification evidence or if the relations occurred in the ChEA database. To not overfit the features toward a small set of high frequency True interactions, we created a list of high frequency known gene–gene interactions during tuning (see Section 2.5) and labeled these candidate relations as Unknown. In contrast, we defined a False candidate relation as a gene–gene candidate relation that was either annotated in the Negatome 2.0 database (Blohm *et al.*, 2014) or was randomly selected with no interaction evidence. For the Negatome database, we used the combined-stringent data set (containing both the manual- and PDB-stringent data sets), further restricted to human interactions. For the random False candidate relations, we selected eight percent of candidate relations that were not annotated in BioGRID or ChEA and did not contain potential interaction evidence as negative examples.

Overall, we used this strategy to mark 42 736 candidate relations as True, 65 606 relations as False and 1 617 806 relations as Unknown. Because the distant supervision can incorrectly annotate a sentence and because we wanted to verify that a labeled sentence contained an interaction, we marked each True and False candidate relation also as Unknown. In this way, DeepDive could assign a candidate relation a probability if that relation was used for training.

To increase the potential number of sentences denoting negative interactions (e.g. *Gene A* does not interact with *Gene B*), we included an additional corpus of 6585 abstracts from PubMed that contained co-occurring genes from the Negatome database. These annotations were only used for training the system and were not used as evaluation.

### 2.4 Inference with DeepDive

DeepDive is a system for probabilistic inference that utilizes factor graphs to compute probabilities for random variables. For our work, we created a random variable is_correct for each candidate relation. We input all candidate relations with is_correct labels into DeepDive, with 50% training examples held-out for learning feature weights as well as internal testing and calibration. Because information regarding a gene–gene interaction may be spread out across multiple sentences in a document, we used conditional random fields to link candidate relations to the previous mention of the gene pair in the document. For high-throughput Gibbs sampling in DeepDive, we used 1000 learning iterations with one sample for each learning iteration, 1000 inference iterations, a diminish learning rate of 0.95 and a learning rate of 0.001. The output included a probability for each candidate relation’s is_correct value being True for a given sentence. On the basis of previous work with DeepDive (Niu *et al.*, 2012a, b; Zhang *et al.*, 2013), we applied a probability cut-off of 0.90 for calling an extraction as correct; all other candidate relations were marked as incorrect.

### 2.5 System calibration

We manually tuned the gene–gene extractor by iteratively updating the features and training examples (Fig. 1D). For each iteration, we conducted an error analysis; we calculated precision and an estimated recall using 100 random candidate relations with a probability greater than 90% and 100 random candidate relations with a probability less than 90%. At the end of each iteration, we updated the extractor and overall system to address missing feature patterns and distant supervision and then reran the extractor and DeepDive to repeat the process. Prior to system development, we decided to freeze the system once the system achieved an estimated precision and/or recall of 90% for a given iteration.

With each iteration of the error analysis, we used the snowball technique to increase positive training examples in the following iteration. We defined the snowball set as the set of gene–gene pairs that were labeled as Unknown candidate relations in the previous iteration but were a true interaction from the text. This set was then added as an additional source of True training examples, along with the BioGRID and ChEA sets. In this way, we learned new informative features based on the sentences that had not been seen by the system during previous iterations.

### 2.6 Evaluation

We evaluated our system against a gold standard of curated PPIs and using random curation of candidate relations. We conducted a document-level evaluation where we selected the gene–gene pair with the highest probability from all sentences containing that pair across the document as the representative interaction. For physical PPIs, we compared against the DIP. We restricted this curation set to be human only, no homodimers and *PLOS Biology* articles. This gold standard contained 226 PPI-document pairs from 71 unique documents. We also curated a random sample of 264 candidate relations with probability ≥90% (Curation_Positive) and 299 candidate relations with probability less than 90% (Curation_Negative) from a set of 10 000 random PLOS documents. For the DIP gold standard, we calculated both precision and recall. For the Curation_Positive, we calculated sentence-level and document-level precision. Sentence-level precision only counted correct candidate relations in a given sentence as true positives. However, sentences do not exist in isolation in the document and thus we also calculated document-level precision where a candidate relation was counted as a true positive if the genes interacted in the document. To not train and tune the system on the documents in the gold standard and curation sets, we removed the 71 PLOS documents from DIP and the 10 000 random documents for the curation until after tuning was complete. We selected the 10 000 documents to provide a large corpus from which to obtain relevant interactions for curation.

### 2.7 Interaction trends

To understand how interaction knowledge changes over time, we calculated interaction trends for each gene that occurred in an interaction. We defined an interaction document as a document that contained at least one interaction extracted by DeepDive. For each gene, we calculated the number of documents from 2004 to 2014 that contained interactions involving that gene. We normalized the number of interaction documents for each gene by the number of total interaction documents per year.

## 3 Results

After the tuning process, we evaluated the extracted gene–gene interactions against the DIP database and against curation of random extractions.

### 3.1 DeepDive extractions and features

**Fig. 3.**
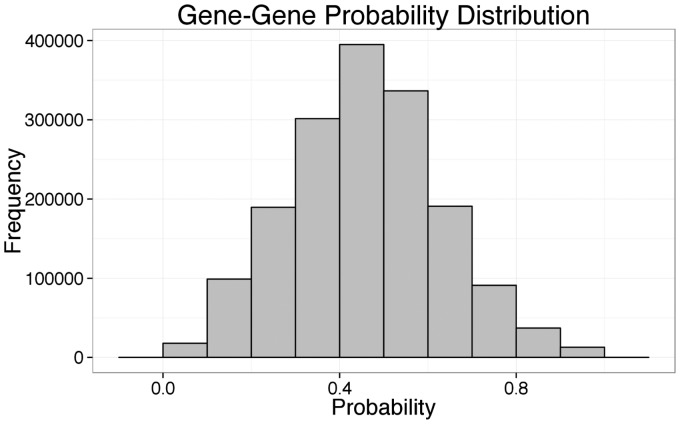
Histogram of probabilities assigned to gene–gene candidate relations

Given the candidate relations from the gene–gene extractor, DeepDive computed a probability that each relation was a true interaction. Out of 1 671 944 total relations not used for training, there were 12 390 gene–gene relations with a probability ≥0.9 and 47 881 relations with a probability ≥0.8. Of these 12 390 gene–gene relations, there were 4182 gene symbol pairs, 5355 NCBI Gene database GeneID pairs and 3356 GeneID pairs with multiple mappings from gene symbols to GeneIDs removed. The full histogram of probabilities for all candidate relations is provided in Figure 3. Additional calibration plots for the held-out test set from DeepDive are depicted in Supplementary Figure S1. Of the 3356 unique GeneID pairs, 2271 (67.7%) were not annotated in BioGRID, 3092 (92.1%) were not annotated in DIP and 2222 (66.2%) were not annotated by either DIP or BioGRID (database overlap for DIP, BioGRID and Negatome available in Supplementary Data). Additionally, three GeneID pairs were annotated by the Negatome database but were unannotated by either DIP or BioGRID. Despite annotation by the Negtome database, all three pairs were correct interactions from their respective sentences and documents.

In total, our system extracted 3 422 176 features, with 724 features with a weight greater than 0.1. Table 1 lists the top 10 positive features constructed from feature patterns for gene–gene relationships (top 724 features are provided in Supplementary Data). A positive weighted feature provides evidence that the factor function with that feature in DeepDive’s factor graphs labels true interactions, while a negative feature provides evidence for negative interactions. Unsurprisingly, the top features describe sentences with the verbs ‘bind’, ‘interact’ and ‘regulate’ between the genes. While the top 724 features contain specific dependency paths between genes in the sentence, a collapsed dependency path of gene—verb—gene had the greatest signal of the dependency path features. Overall, window-based features comprised 67% (487) of the top 724 features. Supplementary Table S1 lists the frequencies for feature patterns present in the top features (feature descriptions provided in Supplementary Data).

**Table 1.** Top 10 positive gene–gene features from DeepDive

Feature	Weight
Single_Verb_Between_Genes_[bind]	1.25
Single_Verb_Between_Genes_[interact]	1.07
Verb_On_Dependency_Path_[bind]	0.91
Verb_On_Dependency_Path_[interact]	0.74
Single_Verb_Between_Genes_[regulate]	0.67
Verb_Between_Genes_[bind]	0.63
Verb_On_Dependency_Path_[regulate]	0.58
Window_Left_Gene1_Phrase_[GENE and]	0.57
Window_Right_Gene2_1gram_[protein]	0.57
Window_Left_Gene1_Phrase_[interaction between]	0.51

### 3.2 Evaluation against DIP

We compared 226 PPIs curated from 71 PLOS documents by DIP to candidate relations from our system with ≥90% probability. Our results are summarized in Table 2. Our system obtained 48% precision and 11% recall (DIP evaluation set). However, 38% of the false positives were true interactions that were unannotated by DIP. With these false positives as true positives, our system obtained 68% precision with a slight boost of recall to 14% (DIP_Rescue). However, when we compare against interactions where both the standard gene symbols (official HGNC or accepted alternate symbol) and the interaction are present in the same sentence, we obtain 46% recall (DIP_Sentence). Finally, we evaluated against DIP with indirect interactions in the false positives as true positives (DIP_Indirect). Our system obtained 76% precision and 49% recall. The interactions that were outside the gene–gene interaction definition included non-standard gene symbols (54%), full names only (17%) and non-human symbols (16%).

**Table 2.** Evaluation against the DIP curated gold standard

	Precision	Recall	*F* measure
DIP	0.48	0.11	0.17
DIP_Rescue	0.68	0.14	0.23
DIP_Sentence	0.68	0.46	0.54
DIP_Indirect	0.76	0.49	0.59

DIP contained all unique interactions, DIP_Rescue included true positives not curated by DIP, DIP_Sentence included DIP_Rescue but only included standard gene symbols co-occurring in a sentence as positives in DIP and DIP_Indirect included DIP_Sentence and indirect interactions as true positives.

### 3.3 Evaluation against curation

In addition to DIP, we evaluated 264 random candidate relations with ≥90% probability (Curation_Positive) and 299 random candidate relations with probability less than 90% (Curation_Negative) against manual curation. Results are summarized in Table 3. For the Curation_Positive, we achieved a precision of 62% when evaluated on direct interactions (Curation_Positive_Stringent) and a precision of 79% on direct and indirect interactions (Curation_Positive_All) for each individual sentence. For document-level interactions, the system achieved a precision of 71% on Curation_Positive_Stringent and 83% on Curation_Positive_All. For the Curation_Negative, there were only four false negatives out of 299 negatives (1.3%). One of these false negatives was a TFI, while another was an interaction found in a reference title with no other mention in the document.

**Table 3.** Sentence- and document-level precision for Curation_Positive set of gene–gene candidate relations

	Sentence-level precision	Document-level precision
Curation_Positive_Stringent	0.62	0.71
Curation_Positive_All	0.79	0.83

The false positives included two categories of errors in the pipeline. The first are non-gene entities (NG), where a gene symbol is the same as an acronym in text. These ambiguous gene symbols included domains (e.g. PTB gene vs. PTB domain) and other general acronyms (e.g. APC gene vs. APC acronym). APC, in particular, is a difficult case due to its widespread use as an acronym for studies, organizations and processes, along with the APC gene symbol being the widely used official symbol for the adenomatous polyposis coli gene. The second category of pipeline errors is bad parse errors. These errors included incorrect sentence splitting and incorrect tokenization from the parser. In the case of sentence splitting, instead of correctly evaluating two genes co-occurring in the same sentence, two or more sentences were combined as a single sentence and the two genes in the candidate relation occurred across these sentences.

### 3.4 Interaction trends

**Fig 4.**
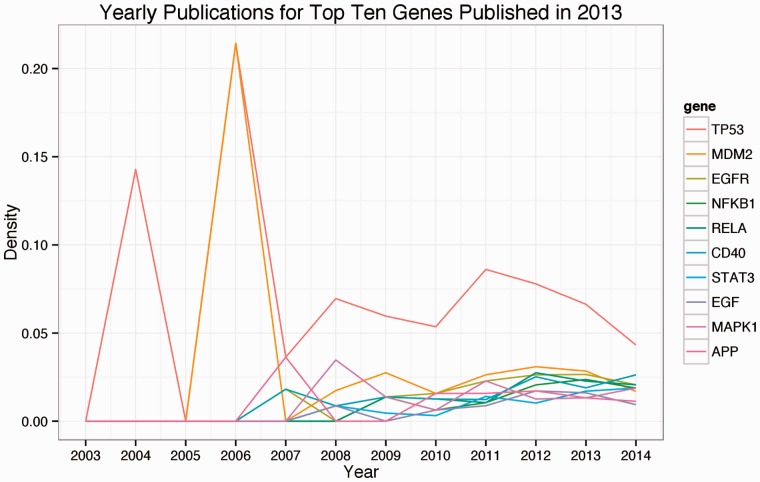
Number of publications per year for genes appearing in high probability interactions. Top genes are ordered by publication count in 2013

By extracting all interactions from three PLOS journals, we were able to detect interaction trends at the journal level and at the gene level. On average in documents that contained at least one interaction, *PLOS Biology* had 4.2 interactions per document. *PLOS One* documents contained 3.2 interactions per interaction document and *PLOS Genetics* contained 2.7 interactions per interaction document. We also evaluated trends of gene-based publications from 2004 to 2014. Figure 4

depicts the number of documents per year for the top 10 genes in 2013.

## 4 Discussion

In this work, we developed a gene–gene relation extractor for the system DeepDive and applied it to the entirety of three PLOS journals. Our system performance is on par, if not better, than current systems that have been applied only to abstracts. We extracted direct physical PPIs, indirect interactions and TFIs to create a complete view of known protein and gene interactions. By extracting both PPIs and TFIs, our work enables not only the construction of protein interaction networks but also how these networks impact or are impacted by gene regulation.

While different groups have focused on extracting physical PPIs, other types of gene–gene relationships, including transcription factors, are key for understanding biological processes, disease etiology and drug response (Lee and Young, 2013). To build a complete knowledgebase of gene and protein relationships, we extracted both physical PPIs as well as transcription factors with their respective target genes. Apart from the impact these different types of interactions have on one another, oftentimes a single isolated sentence will be too vague to deduce the type of interaction. For example, the verb ‘associate’ can be used for both PPIs and TFIs and does not provide enough information to specify the interaction type. Evaluating a combined interaction system presents a challenge because there is no combined test set for both types of gene–gene interactions. To properly evaluate, we checked extractions in DIP-curated documents for missed transcription factor relationships and counted them as true positives. Additionally, we performed a random curation to assess performance for both types of interactions.

By using the entirety of *PLOS Biology, PLOS Genetics* and *PLOS One*, we showed how scientific knowledge about a protein’s interactions change over the course of a decade. As the PLOS family of journals is just over a decade old, the majority of documents containing interactions occurred after 2008. With a larger corpus across older journals, we will be able to show how genes grow or wane in popularity due to recent research. In the future, general relation extraction on full-text journals spanning a decade or more will allow for more nuanced analyses about how our understanding on these relations changes over time.

We constructed individual binary features from sentences encountered in the training data that matched high-level feature patterns. As this strategy created millions of individual features, we were concerned with overfitting the feature weights to the training data. However, the tuning process highlighted sources of overfitting that were subsequently removed from the system on the next iteration. These sources of overfitting included highly frequent training examples that were removed from the training data. After tuning, 93.9% of the features had a weight less than 0.001 (3 212 458 out of 3 422 176) and did not impact the final probability of a given candidate relation. The remaining features were not only learning patterns in a noisy training set but were also learning patterns for gene co-occurrence due to shared biological processes that are not strictly interactions.

DeepDive allows users to automatically annotate candidate entities or relations with distantly supervised labels to learn underlying patterns among the input features. By distantly supervising the training labels, we do not curate each sentence to confirm that the two genes are interacting based on the sentence. Rather, we label over 100 000 candidate gene–gene relations as training examples even though most will be co-occurrence. Using these uncertain training examples, we were able to detect true interaction patterns at the sentence level.

Instead of providing a binary output or label for each candidate, DeepDive provides a probability that the candidate entity or relation has some attribute (e.g. is_correct). Thus, we can select a probability cut-off to call a given output a specific label. Additionally, DeepDive is specifically calibrated to have accuracy correlated with probability. The system calculates accuracy using the distant supervision training labels, and thus this calibration represents DeepDive’s performance based on the provided labels. During tuning, the user may discover that the distant supervision labels are missing an entire class of relations and thus incorrectly labeling candidates. For example, if one only uses random English word pairs as a False relation, then the system may incorrectly say all co-occurring genes are true relations due to random gene–gene pairs existing with an easily recognizable genetic context that differs from random English words.

DeepDive requires several iterations of tuning to remove noisy distant supervision gene pairs and add or remove feature patterns. At the end of each iteration of DeepDive, we conducted an error analysis to identify sources of error in the system. We completed 24 in-depth error analyses before freezing the system for evaluation. The false positives and false negatives from these error analyses were caused by unbalanced feature weights and training examples, lack of training examples for a given feature (low weighted feature) and missing features. Important to note, we did not aim to fix specific candidate relations but rather, to identify either missing or incorrectly weighted feature patterns.

DeepDive’s internal calibration and the user calibration are two distinct calibration steps with respect to machine learning. For the system calibration (learning features to predict labels), DeepDive holds out a test set from the training labels to evaluate DeepDive’s predictions based on the input data. On the other hand, user calibration refers to the process of adding and/or refining features or training labels manually. DeepDive’s internal calibration plots are shown in Supplementary Figure S1. Plot (a) shows accuracy for the held-out test set at different probability cut-offs. This calibration depicts DeepDive’s performance solely based on the provided training labels. On the basis of prior work with constructing features, we only required 24 complete iterations of user calibration (calculating an estimate of precision and recall on sample sentences) before we froze the system. To decide when to stop this user calibration, we decided on an estimated precision and/or recall of 90% for a given iteration.

Because our system ultimately extracts interactions from an input corpus, we cannot directly compare on previously constructed interaction data sets for text mining. This comparison would require new input documents, which may alter results. We evaluated our system against the DIP interaction database; however, this evaluation has its limitations. One limitation is that the 71 documents were solely from *PLOS Biology*, but the system was largely trained on *PLOS One* documents. Additionally, the DIP set contained PPIs curated from documents with experimental evidence that may not be well represented in our overall PLOS corpus.

There have been multiple papers and reviews focusing on protein interaction method comparison; however, we chose not to directly compare to other methods for multiple reasons. First, many of these methods were designed for abstracts only and may not perform as well on full-text articles due to different levels of detail in the full text compared with the abstract. Second, these methods were tested on many different corpora and small-scale gold standard data sets. In one comparison performed by Tikk *et al.* (2010), performance metrics vary depending on the gold standard used. In addition, some methods, such as PIE (Kim *et al.*, 2012), aim to prioritize protein interaction abstracts or articles.

Our system has multiple components including text preprocessing, gene extraction, feature construction and distant supervision. A false positive or negative can arise from any of these components; however, we were interested in the number of false positives from the text preprocessing and gene extraction stages. An error in either of these two categories represents an impossible interaction in our system: one or both gene symbols actually being an acronym for a non-gene entity (NG) or two genes not occurring in the same sentence (NP). As we wanted the highest recall for gene symbols and as these symbols are largely known and stored in databases such as NCBI Gene database and UniProt, we decided to find initial genes with a dictionary-based approach and have the system learn that a non-gene entity cannot be in a gene interaction. However, we discovered numerous cases where authors only referred to genes by their full names or only used non-human and non-standard gene names. To address these errors, we could combine a dictionary-based approach along with a named entity recognition tool such as Banner (Leaman and Gonzalez, 2008) or GenNorm (Wei and Kao, 2011) (or even DeepDive) to catch these non-standard gene name variants. Sentence parsing errors occurred when two or more sentences were concatenated together, with two genes extracted as a single candidate relation even if the genes were present in separate sentences. These errors were introduced at the text preprocessing stage. One potential solution would be to train the Stanford parser on biomedical text to learn the different forms of sentences, tokens and parts of speech when complex gene names, symbols, acronyms and unknown words are frequently used. Alternatively, we can apply the GENIA Tagger (Tsuruoka *et al.*, 2005) to tokenize and label words with parts of speech; however, this would not address incorrect sentence splitting.

To investigate errors occurring at the extractor stage, we can disregard these NG errors and improve precision roughly five points for Curation_Positive_Stringent and Curation_Positive_All to 66% and 84%, respectively. Disregarding the NP errors, precision for Curation_Positive_Stringent and Curation_Positive_All increased two points to 64% and 81%, respectively. Removing both the NG and NP errors and focusing only on extractor errors, the system achieved 68% and 86% precision for Curation_Positive_Stringent and Curation_Positive_All, respectively.

Text mining methods and applications require access to either abstracts or full-text articles for data extraction. While researchers have access to the majority of biomedical abstracts through NCBI’s PubMed database, access to full-text articles remains limited. As of July 2015, there are over 3.5 million articles from nearly 5000 journals in PubMed Central (PMC) but only 646 681 available through PMC’s Open Access Subset. This set includes many journals that are not relevant for mining gene–gene interactions but may still mention co-occurring genes. To reduce noise from journal selection, we focused on relevant journals from a single publisher. It is important to note that these three journals comprise almost 17% of PMC’s Open Access journal articles. Future system tuning on additional open access journals will provide more interactions for use in the research community.

DeepDive contains internal parallelization and optimization to efficiently handle large document corpora. We set DeepDive’s parallelization parameter to run 48 processes with a maximum of 64 GB of memory. Not including document parsing with Stanford CoreNLP, runtime was less than 2 h to load documents into the database, construct candidate mentions with features and run the inference engine.

In this work, we show the utility of DeepDive and distant supervision for extracting gene–gene interactions from a large corpus of documents. While we tuned the system for three PLOS journals with over 100 000 full-text documents, adding additional documents or abstracts would require several rounds of system tuning. Our work is the first application of DeepDive to the biomedical domain. Specifically, we extracted 12 390 gene–gene relations linked to sentences from over 100 000 full-text documents from three PLOS journals. These relations are available in Supplementary Data. This work enables an improved and more precise understanding of gene and protein interactions within the cell to further both experimental and computational research.

## Supplementary Material

Supplementary Data
