# Supplementary material for: Large-scale extraction of gene interactions from full-text literature using DeepDive
Source: Bioinformatics. 2015 Sep 3;32(1):106–13. doi: 10.1093/bioinformatics/btv476 (PMC4681986; doi:10.1093/bioinformatics/btv476)
Supplement: Supplementary Data [file supp_32_1_106__index.html]

Large-scale extraction of gene interactions from full text literature using DeepDive — Large-scale extraction of gene interactions from full-text literature using DeepDive — Large-scale extraction of gene interactions from full-text literature using DeepDive — Supplementary Data 

# Large-scale extraction of gene interactions from full-text literature using DeepDive

## Supplementary Data

files

- Supplementary Data - zip file
