# Supplementary material for: Large-scale extraction of gene interactions from full-text literature using DeepDive
Source: Bioinformatics. 2015 Sep 3;32(1):106–13. doi: 10.1093/bioinformatics/btv476 (PMC4681986; doi:10.1093/bioinformatics/btv476)
Supplement: Supplementary Data [file supp_btv476_suppl_data.zip › Supplementary_File_Descriptions_rev.pdf]

Supplementary File Descriptions

1. 12,390 Gene-Gene Extractions above 90% Probability

File: Supplementary_Data_GeneGene_Extractions.xlsx

Columns:
1 – journal
2 – article_id: article id from PLOS, including both journal and article identifier.
3 – pubmed_id: NCBI PubMed identifier, if exists
4 – sentence_id: sentence number from Stanford CoreNLP 1.3.4
5 – mention1_offset: word offset using tokenization from Stanford CoreNLP for mention1
6 – mention2_offset: word offset using tokenization from Stanford CoreNLP for mention2
7 – mention1: text representation in sentence of mention 1 in the interaction
8 – mention2: text representation in sentence of mention 2 in the interaction
9 – geneids1: NCBI GeneID mapping for mention 1
10 – geneids2: NCBI GeneID mapping for mention 2
11 – probability: probability of interaction from DeepDive

2. 724 Top Features

File: Supplementary_Data_Top_Features.txt

Columns:
1 – description – gene-gene feature
2 – weight – weight for the feature from DeepDive

Feature patterns in the top 724 features:

DEP_PAR_VERB_CONNECT_with[*verb*]	A verb (*verb*) appears on the dependency path between two gene mentions
NEG_VERB_BETWEEN_with[*verb*]	A negated verb (*verb*) appears in the words between two gene mentions
PREP_PATTERN[*phrase*]	Two gene mentions are found in a prepositional interaction pattern with *phrase*
SINGLE_VERB_BETWEEN_with[*verb*]	Only one verb (*verb*) appears in the words between two gene mentions
VERB_BETWEEN_with[*verb*]	Verb (*verb*) appears in the words between two gene mentions. Other verbs exist in the word sequence.
WINDOW_*X*_M*Y*_*Z*_with[*word*]	Single word (*word*) 1 or 2 (*Z*) words right or left (*X*) of gene mention (*Y*). If word is GENE, then the word is a gene symbol.
WINDOW_*X*_M*Y*_PHRASE_with[*phrase*]	2 word phrase (*phrase*) right or left (*X*) of gene mention (*Y*)
WORDS_BETWEEN_with[*phrase*]	Word sequence (*phrase*) between two gene mentions
WS_3_GRAM_with[*phrase*]	3 word phrase (*phrase*) within the word sequence between two gene mentions
GENE_M*X*_FOLLOWED_BY_PLURAL_NOUN_with[*word*]	Gene mention (*X*) is followed by the plural word (*word*)
DEP_PAR[*path*] or ROOT_'*path*'	The dependency path (*path*) between two gene mentions.

Boolean Patterns

GENE_FOLLOWED_BY_DOMAIN_WORD	One of two gene mentions followed by domain word (e.g., domain, motif)
NEG_SECOND_GENE	Negation immediately before second gene mention in the sentence

3. 3356 Unique GeneID Pairs Mapped to Databases

File: Supplementary_Data_GeneGene_Extractions_Database.txt

Note: GeneID pairs ordered for each unique interaction

Columns:
1 – GeneID1: NCBI GeneID mapping for one gene in interaction
2 – GeneID2: NCBI GeneID mapping for other gene in interaction
3 – Symbol1: Official gene symbol from NCBI for GeneID1
4 – Symbol2: Official gene symbol from NCBI for GeneID2
5 – DIP: Presence of interaction in DIP database
6 – BioGRId: Presence of interaction in BioGRID database
7 – Negatome: Presence of interaction in Negatome database
