# Supplementary material for: Large-scale extraction of gene interactions from full-text literature using DeepDive
Source: Bioinformatics. 2015 Sep 3;32(1):106–13. doi: 10.1093/bioinformatics/btv476 (PMC4681986; doi:10.1093/bioinformatics/btv476)
Supplement: Supplementary Data [file supp_btv476_suppl_data.zip › Supplementary_Tables_Figures_rev.pdf]

Supplementary Tables and Figures

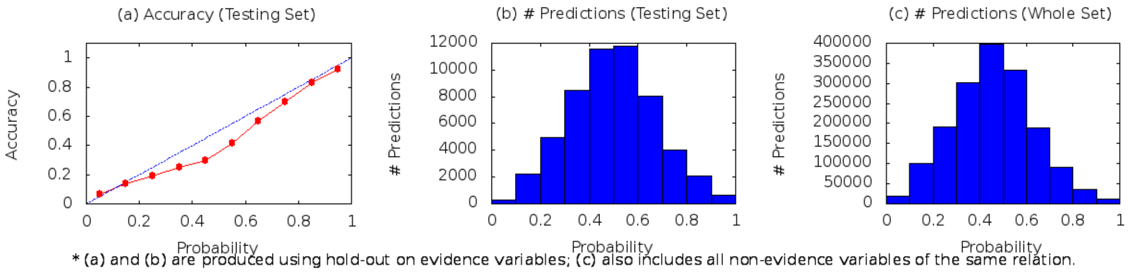


**Fig S1.** Calibration plots from DeepDive. Variable refers to the Gene-Gene candidate mentions. Plot (a) depicts accuracy at different probability cut-offs for the held-out test set. Plots (b) and (c) are histograms for different probability buckets on the held-out test set and whole set, respectively.

**Table S1.** Number and frequency of feature patterns in the top 724 Gene-Gene features.

| Feature Pattern | Number of features | Frequency |
|---|---|---|
| WINDOW_*X*_M*Y*_*Z*_with[*word*] | 312 | 0.430939227 |
| WINDOW_*X*_M*Y*_PHRASE_with[*phrase*] | 175 | 0.241712707 |
| WS_3_GRAM_with[*phrase*] | 50 | 0.069060773 |
| VERB_BETWEEN_with[*verb*] | 46 | 0.063535912 |
| DEP_PAR[*path*] or ROOT_'*path'* | 38 | 0.052486188 |
| WORDS_BETWEEN_with[*phrase*] | 30 | 0.041436464 |
| DEP_PAR_VERB_CONNECT_with[*verb*] | 27 | 0.037292818 |
| SINGLE_VERB_BETWEEN_with[*verb*] | 22 | 0.03038674 |
| GENE_M*X*_FOLLOWED_BY_PLURAL_NOUN_with[*word*] | 15 | 0.020718232 |
| PREP_PATTERN[*phrase*] | 4 | 0.005524862 |
| NEG_VERB_BETWEEN_with[*verb*] | 2 | 0.002762431 |
